# MicroED and its Impact on Form Space Elucidation

**DOI:** 10.1063/4.0001079

**Published:** 2025-10-27

**Authors:** Roger D Sommer

**Affiliations:** Bristol Myers Squibb, New Brunswick, NJ, USA

## Abstract

Understanding the solid form landscape of pharmaceuticals is crucial to the success of product development. Crystallization and crystal structure determination play a central role in this endeavor. While single-crystal X-ray diffraction is a go-to technique for insight to the solid state, it depends on the availability of sufficiently large crystals for the experiment. The introduction of micro electron diffraction to this effort offers expedient access to structural information and decreases the crystal size parameter for structure determination. This talk will share how the interplay of micro electron diffraction, single-crystal X-ray diffraction, and other techniques provides elucidation of the form space of new drug substances.

